# Characterization of nanoplastics and small-sized microplastics in sewage treatment

**DOI:** 10.1038/s41598-025-15504-9

**Published:** 2025-08-17

**Authors:** Shaima Iskandarani, Sarvajith Manjunath, Rosa Busquets, Lorenzo Raeli, Pascal E. Saikaly, Luiza C. Campos

**Affiliations:** 1 Centre for Urban Sustainability and Resilience, Department of Civil, Environmental and Geomatic Engineering, University College London, Gower St, Bloomsbury, London, WC1E 6BT UK; 2 Biological and Environmental Science and Engineering (BESE) Division, King Abdullah University of Science and Technology (KAUST), Thuwal, 23955-6900 Kingdom of Saudi Arabia; 3 Faculty of Health, Science, Social Care and Education, School of Pharmacy and Chemistry, Kingston University, Penrhyn Road, Kingston upon Thames, KT1 2EE UK; 4 Environmental Science and Engineering Program, BESE Division, King Abdullah University of Science and Technology (KAUST), Thuwal, 23955-6900 Kingdom of Saudi Arabia; 5 Bioscience Core Lab, KAUST, Thuwal, 23955-6900 Kingdom of Saudi Arabia

**Keywords:** Wastewater, Treated sewage effluent, Sewage treatment plant, Nano-flow cytometry, Confocal Raman, Chemical engineering, Civil engineering, Analytical chemistry, Environmental chemistry

## Abstract

The presence of nanoplastics (NPs) in sewage treatment plants (STPs) remains a critical yet underexplored environmental issue. Here, we present a novel investigation into the occurrence, recovery, and characterization of nanoplastics and small-size microplastics (50–2500 nm) in raw and treated sewage effluent from a full-scale STP (treating 4000 m^3^/day) operating with activated sludge. To our knowledge, this research includes the first confirmed assessment of nanoplastics in such a system and applies nano-flow cytometry to wastewater analysis for the first time globally. It is also the first study addressing micro- and nanoplastics in wastewater in Saudi Arabia, advancing plastic particle analysis in complex matrices. Particles in the 50– < 100 nm range accounted for 44% of total particles detected in STP effluents. Overall, plastic particles accounted for 16% (± 10%) of total particles within 50-2500 nm in raw sewage, increasing to 41% (± 13%) in treated effluent. This increase highlights the inefficiency of conventional treatment in fully removing plastic particles and suggests preferential removal based on size or density. The composition of a representative selection of particles was characterized by micro-Raman spectroscopy and Scanning Electron Microscopy with Energy-Dispersive X-ray. Identified polymers included polystyrene, polyvinyl chloride, polyethylene, polytetrafluoroethylene, polyamide, and polypropylene. These findings provide important insights into treated sewage composition, particularly for reuse in arid regions.

**Supplementary Information:**

The online version contains supplementary material available at 10.1038/s41598-025-15504-9.

## Introduction

In recent years, there has been an increase in global concern about plastic pollution due to its widespread distribution, persistence, and environmental impact^1^. Microplastics (MPs) and nanoplastics (NPs) are categorized based on their size. For clarity and consistency, this study adopts definitions proposed by the International Standard Organization (ISO). Accordingly, MPs are plastic particles ranging from 1000 nm to 1 mm, and large MPs are those within 1–5 mm (ISO 24187:2023^2^). NPs are plastic particles with at least one external dimension between 1 and 1000 nm (ISO/DIS 21362^3^). Recent policy analyses have indicated that the absence of harmonised legal thresholds continues to hamper the regulation of plastic particles^4^.

Definitions aside, plastic breaks down into particulates that are detected in various environmental compartments and biological systems^5^. Moreover, some plastic particles can infiltrate the food web, bioaccumulate^6^, and pose risks to humans and the environment^7,8^. MPs larger than 5000 nm were identified in human organs, such as the lungs, intestines^9^, and placenta^10^. NPs, in particular, raise concerns due to the toxicity derived from their nano-scale size, which allows them to cross biological barriers, such as the blood–brain barrier (particles ~ 293 nm)^11^. Several recent high-impact studies show that the NPs carried by such effluents can move through food webs and breach critical physiological barriers. A recent toxicological review demonstrates that intracellular NPs can induce DNA damage and neurotoxicity across multiple cell types^12^, underscoring potential human-health hazards. Another human autopsy study echoes this risk by observing polyethylene fragments 100–200 nm in size accumulating in the frontal cortex at concentrations 7–30 times higher than in the liver or kidney, confirming systemic distribution to the brain^13^. Moreover, agricultural exposure routes are equally plausible; hydroponic and soil-pot experiments show that ≈ 80–100 nm NPs taken up by roots can translocate to edible leaves of lettuce and pak-choi, establishing a direct dietary pathway^14,15^. Also, a recent mesocosm study demonstrated that polystyrene (PS) NPs at concentrations as low as ~ 21 µg/L can disrupt freshwater food webs, with stronger effects observed at 214 µg/L and above^16^. These findings provide early ecotoxicological benchmarks and highlight the urgency of defining safe environmental thresholds for NPs, especially in the context of wastewater reuse. Clearer dose–effect relationships of NPs will emerge as more studies are published.

Sewage treatment plants (STPs) can be a source of plastic pollution. The untreated sewage and treated sewage effluents (TSE) act as conduits for plastic debris reaching the ocean via rivers when treatment processes fail to intercept them effectively^17,18^. Plastics in TSE include microfibers, fragments, beads, and films. These particles are typically < 100 μm^19–21^. MPs in wastewater and their release into aquatic environments have been studied, however the occurrence and release of NPs from STPs remain unexplored due to analytical difficulties. Moreover, pilot-scale polishing trains that couple tight-pore ultrafiltration, nanofiltration, or reverse-osmosis with secondary effluent remove > 90%, often > 99%, of model 50–100 nm NPs^22^. However, even these high-pressure barriers allow a residual tail < 100 nm to permeate^23^. To date, no full-scale wastewater treatment line has achieved complete retention of this sub-100 nm fraction, underscoring a critical technology gap for future water-reuse schemes^24^.

Previous studies have primarily focused on the removal of > 1000 nm MPs, often reporting high removal efficiencies^25–27^. A major source of MPs in wastewater is synthetic fibres shed from textiles during laundering, which are released into greywater and enter wastewater systems^28^. Due to limitations in conventional treatment processes, a significant portion of these fibres remains untreated and are discharged into the environment^29^. Recent studies estimate that a single wash can release up to 700,000 fibres, many of which escape removal in conventional treatment plants and are discharged with the effluent or accumulate in sewage sludge^30,31^. Additionally, plastics in sewage may undergo fragmentation into smaller particles, including NPs, during physical (e.g., mechanical forces), biological (e.g., biodegradation), or chemical (e.g., UV treatment) processes within STPs^28,32^. Shear stress forces, such as those encountered in turbulence and mixing processes in STPs, can cause propagation and surface erosion^33^. This internal fragmentation is underreported and highlights the dual role of STPs as both receivers and generators of plastic debris. Particle size plays a crucial role in the dispersion of plastic particles, with smaller particles (< 300,000 nm) being more readily dispersed in aquatic environments compared to larger particles^34^. Although data on NPs are not yet widely available, they are more concerning ecotoxicologically since they are understood to be greater hazards for aquatic ecosystems. Long-term reuse pathways also create a terrestrial sink: after two decades of tertiary-effluent irrigation, orchard soils contained 329 ± 140 MP kg⁻^1^ in the 0–10 cm layer, almost double the 169 ± 47 MP kg⁻^1^ found in nearby rain-fed controls^35^. Biennial sampling of grassland plots over 25 years shows that repeated sewage-sludge applications raise soil MPs inventories by 723–1445% and that these elevated levels persist for at least 22 years^36^. Although such loading can drive particles down to 40 cm depth, hydrothermal (thermal-hydrolysis) pretreatment has been shown to reduce MP concentrations in sludge by up to ~ 80%. Subsequent anaerobic digestion contributes marginally to the reduction of petroleum-based MPs but may aid in breaking down biodegradable plastics^37^.

Flow cytometry has started to be used to analyse MPs in various types of samples such as spiked laboratory solutions, drinking water samples^38^, surface water^39^ and blood^40^. However, despite its advantages, conventional flow cytometry can be affected by low sensitivity and resolution needed to effectively differentiate MPs and NPs from background noise below 1000 nm^41^. The small size, diverse chemical compositions, and potential aggregation of NPs pose significant challenges, often leading to false positives or inaccurate quantification. Additionally, the presence of organic and inorganic contaminants in wastewater can interfere with fluorescence-based detection, further limiting its reliability^42^. To address these limitations, this study, for the first time, to the best of our knowledge, utilises nanoflow cytometry combined with Nile Red staining as an advanced approach for detecting and quantifying NPs and MPs (50–2500 nm) in STPs. The specific objectives of this study are to (1) assess NP abundance and size distribution in untreated and treated sewage effluent, (2) evaluate the efficiency of STPs in removing small size MPs and NPs during treatment processes, and (3) determine whether biological treatment contributes to NP formation or effectively captures existing NPs. To investigate this, the activated sludge process was selected as the focus of this study, as it is the most widely used sewage treatment technology globally.

## Materials and methods

### Sampling

**Fig. 1:**
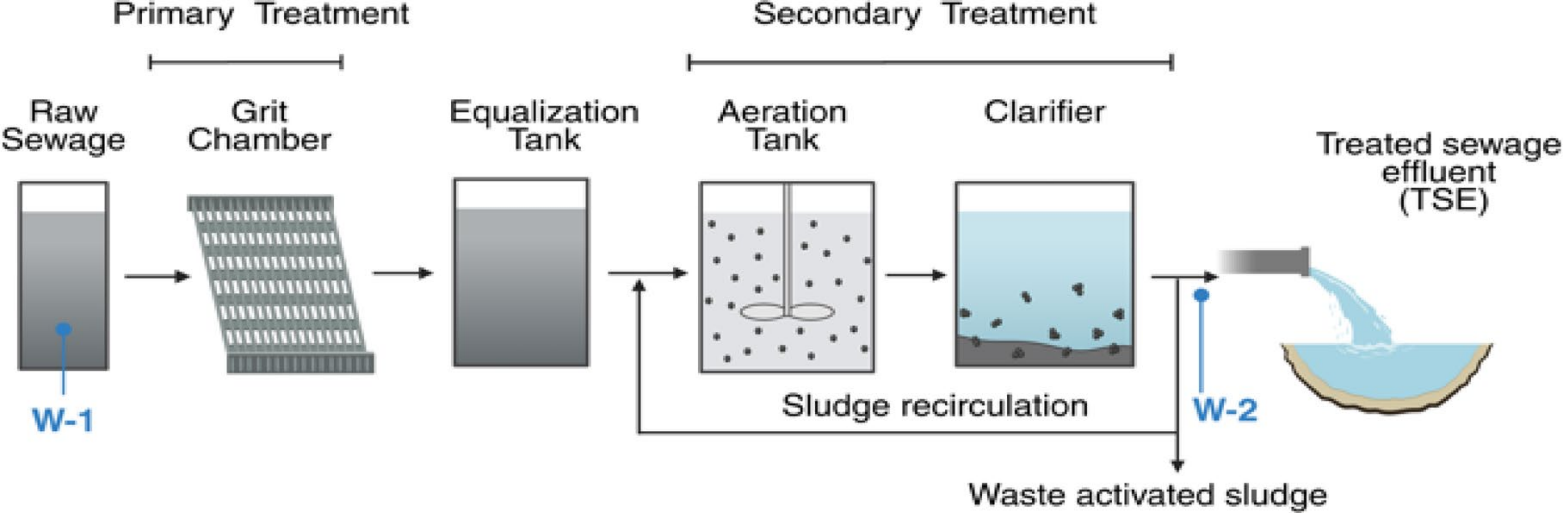
Scheme of the STP based on the activated sludge process. W-1 and W-2 refer to the sampling points.

The samples for NP analysis were collected from an activated sludge STP located in the Kingdom of Saudi Arabia. The STP receives domestic wastewater with an average daily flow rate of 4000 m^3^/d. The schematic of the treatment process is presented in Fig. 1. Three separate sampling events were conducted, each separated by a month. The samples were collected between January and March 2024. The weather conditions during the sampling period were typically sunny and dry, with temperatures exceeding 22 °C. Samples (2 L) were collected in glass bottles from specific locations in the STP: raw sewage before treatment (W-1) and TSE (W-2) (Fig. 1). Only metal or glassware was used during the sampling. At least three replicate samples were collected in glass jars, covered with foil (instead of using a plastic lid), transported to the laboratory, and processed immediately. The characteristics of sewage and TSE are presented in Table 1.

**Table 1:** Characteristics of sewage and treated sewage effluent (TSE).

	Sewage	Treated sewage effluent (TSE)
Chemical oxygen demand (COD)	200–340	30–60
Biological oxygen demand_5_ (BOD_5_)	70–160	< 30
Ammonium-N	46–54	< 0.1
Nitrate- N	0.2–0.8	21–28
Total nitrogen (TN)	47–56	22–31
Phosphate-P	4–5.5	4.1–4.4

All parameters are expressed in mg/L.

### Recovery of NPs

The NPs were extracted following the protocol developed elsewhere^43^ with optimisations to suit the sample type and ensure gentle mixing and controlled heat exposure (60 °C). This allowed visible degradation of organic matter without excessive bubble formation. In brief, 10–20 mL of 30% (v/v) hydrogen peroxide (H_2_O_2_, Sigma Aldrich, St. Louis, MO, USA) was added to 0.5–1 L of raw sewage or TSE samples. Samples were digested at 60 °C on a heat block with continuous magnetic stirring at 60 rpm, until the volume was reduced to 50 mL. To prevent degradation of plastic particles and maintain effective digestion, H_2_O_2_ was added incrementally every 15 min. The digestion temperature of 60 °C was deliberately chosen to remain well below the thermal transition points of the polymers analysed to avoid any risk of degrading polymers. For consideration, Higher Density Polyethylene (HDPE) melts at approximately 133 °C, isotactic polypropylene at 160–165 °C, and PS softens only above its glass transition temperature of 100 °C, melting around 240 °C. Therefore, the digestion conditions were sufficiently mild to avoid any thermal degradation or deformation of the plastic particles.

A representative image (Figure S1 in Supplementary Information) shows that the digested samples were visibly clear prior to staining. This confirms that organic material was largely removed, supporting the reliability of downstream plastic detection. After digestion and cooling to room temperature, 5 mL of a saturated solution of ZnCl_2_ (98% purity, from Thermo Scientific, Madrid, Spain) was added to reach a final density of 1.5 g/mL. This density is sufficient to separate most common plastic types, such as PS, PE and polypropylene (PP)^20,44^. The mixture was then centrifuged at 5000 rpm for 5 min^45^ and the resulting supernatant, potentially containing plastic particles, was poured and filtered.

To prevent the clogging of filters due to MPs, large pore-sized filters were used at the beginning. To separate MPs from NPs, the samples were first filtered through a 50,000 nm metal sieve (200 mm diameter) to remove larger debris. Next, a 2500 nm cellulose acetate filter (90 mm diameter; Millipore, Burlington, MA, USA) was used to retain MPs while allowing smaller particles, including potential NPs, to pass through. The filtrate from the 2500 nm filter, which might contain NPs, was further passed through a series of membrane filters with reducing pore sizes of 450 nm, 200 nm, and 100 nm (47 mm diameter; Millipore, Burlington, MA, USA, and Nucleopore, Maidstone, UK). Each fraction resulted in particle size ranges (in a sequence of filtration, min.-max.) 450– < 2500 nm (retained on the 450 nm filter), 200– < 450 nm (retained on the 200 nm filter), 100– < 200 nm (retained on the 100 nm filter), < 100 nm (remaining liquid portion). The obtained NPs on the filter papers were carefully transferred using forceps to aluminium foil-wrapped Petri dishes, dried at room temperature, and stored for further analysis.

### Identification and quantification of NPs

Confocal Raman spectroscopy (WITec, Oxford Instruments, Ulm, Germany) was used to identify the recovered plastic particles onto filter paper. A 532 nm laser, a grating of 1800 g mm^−1^, and objectives ranging from 200× to 1000× magnification, with an accumulation time of 5 s, were used. The spectra were collected with a resolution of 5 cm^−1^ over the 500–4000 cm⁻^1^ range. Raman spectroscopy conducted on the filtered particle fractions provided chemical confirmation of representative particles within each size range detected by nanoflow cytometry (BIGFOOT, Thermo Scientific, USA). Baseline correction and noise reduction were applied to all spectra before analysis. Polymer identification was performed using the ST Japan 6.1 Raman spectral library. Spectral matching was based on the Hit Quality Index (HQI), where values > 80 were considered indicative of a reliable match. Spectra that did not meet the confidence thresholds were classified as unidentified.

Liquid samples were diluted in ultrapure water obtained with a Milli-Q® Reference system by Merck Millipore (MilliporeSigma, Burlington, MA, USA) and filtered with cellulose acetate filters. Plastic particles were stained by adding 3 µL of a 1 mg/mL Nile Red stock solution in 20% dimethyl sulfoxide to 1 mL of the sample, resulting in a final concentration of approximately 3 µg/mL. The mixture was incubated for 15 min in the dark at room temperature (22–25 °C). Each sample (2 mL suspension) was analysed without a rinsing step to minimise particle loss and maintain the integrity of the original size distribution. Two simultaneous thresholds were set: one for the yellow-green laser (561 nm wavelength) detector channel, targeting the Nile Red emission wavelength (615/15 nm band-pass filter), and another for the violet laser forward scatter channel for Small Particle Detection (SPD) (405 nm—SPD). These thresholds were applied to distinguish plastic particles from background noise generated by dark current, salt crystals, non-plastic particles, and non-specific staining. A dye-only control (Nile Red in filtered Milli-Q water) analysed under identical settings confirmed that the gating removed events arising solely from the dye; residual background was ≈ 4%.

Moreover, to address potential background signal interference, particularly from biological particles or inorganic material of similar size to the analysed MPs and NPs, we implemented a dual-parameter detection strategy. The physical parameter SPD-Forward Scatter (FSC) could capture a narrow range of particles based on size, excluding the larger aggregates at the high-end, and the electronic background noise at the low-end. However, due to overlapping scatter profiles, biological particles of similar size (as well as inorganic materials such as calcium carbonate and silica) could not be confidently distinguished using SPD-FSC alone, as these substances commonly generate significant light scattering and contribute to background signals in complex matrices. To resolve this, a fluorescence threshold was applied to the Nile Red channel, which selectively detects particles containing neutral lipids or hydrophobic polymers. Importantly, the emission wavelength selected for Nile Red was tuned to detect neutral lipids^46^, which are expected to be absent in oxidized biological matter due to H_2_O_2_ treatment, and/or plastic particles. Furthermore, Nile Red shows strong yellow-gold fluorescence in the presence of neutral lipids, while it emits red fluorescence when associated with more polar lipids^46–48^ The optical system used in the Thermo Fisher Bigfoot collected yellow and orange light (616 nm, corresponding to neutral lipids) ignoring the red light (> 650 nm, corresponding to polar lipids). Polar lipids are the only ones that could be present in the sample after the oxidation process due to the H_2_O_2_ treatment (see Section “Recovery of NPs”) prior to cellulose acetate filtration. This dual gating approach, combining SPD-FSC with the more selective Nile Red fluorescence, ensured that only Nile Red-bound plastic particles were included in the analysis, thereby enhancing specificity and effectively reducing interference from both biological and inorganic background components such as calcium carbonate and silica.

The double threshold and voltage settings for these channels were adjusted to ensure that no events were detected when analysing unstained ultrapure water and that only 1–5 events per second were recorded when analysing unstained samples. A weak, non-specific fluorescent signal was observed from Nile Red alone. Therefore, both the sample and ultrapure water controls underwent identical preparation steps, including nanofiltration, and were stained. The controls exhibited a higher background fluorescence in the Nile Red emission channel.

The fluorescence signal detected in the ultrapure water control was used as a reference to differentiate plastic-specific signals (positive) from background noise (negative). These background control experiments were essential for validating the specificity of Nile Red in complex environmental matrices. The fluorescent probe Nile Red is widely employed for MP staining^49^, and it is also commonly used for lipid detection in cytological applications. Although biological particles of similar size overlapped in scatter signals in SPD-FSC, only lipid-containing particles exceeded the Nile Red fluorescence threshold. Notably, the selected Nile Red emission wavelength targeted plastics and neutral lipids, which are unlikely to persist after H₂O₂ oxidation, minimising signal overlap. The dual gating strategy, combining SPD-FSC and Nile Red fluorescence, ensured detection was limited to dye-bound plastic particles.

Moreover, the repeatability of nanoflow cytometry measurements in residual water matrices is greatly influenced by matrix characteristics, particularly when comparing domestic and industrial wastewater. Domestic wastewater, primarily from municipal sources, generally shows moderate to good repeatability (coefficient of variation [CV] ~ 10–20%) due to its consistent composition, rich in organic matter, biosolids, and MPs, and lower concentrations of interfering agents like surfactants and solvents^50^. In contrast, industrial wastewater shows highly variable repeatability, contingent on its origin (e.g., textile, chemical, pharmaceutical, or metal-processing industries). These matrices often contain complex and interfering components, such as high ionic strength, abrasive particles, metal ions, and fluorescent contaminants, which can compromise both light scatter and fluorescence-based detection^51^. Without rigorous pretreatment (e.g., filtration, dilution, desalting, and surfactant removal), repeatability is frequently poor (CV > 30%).

**Fig. 2:**
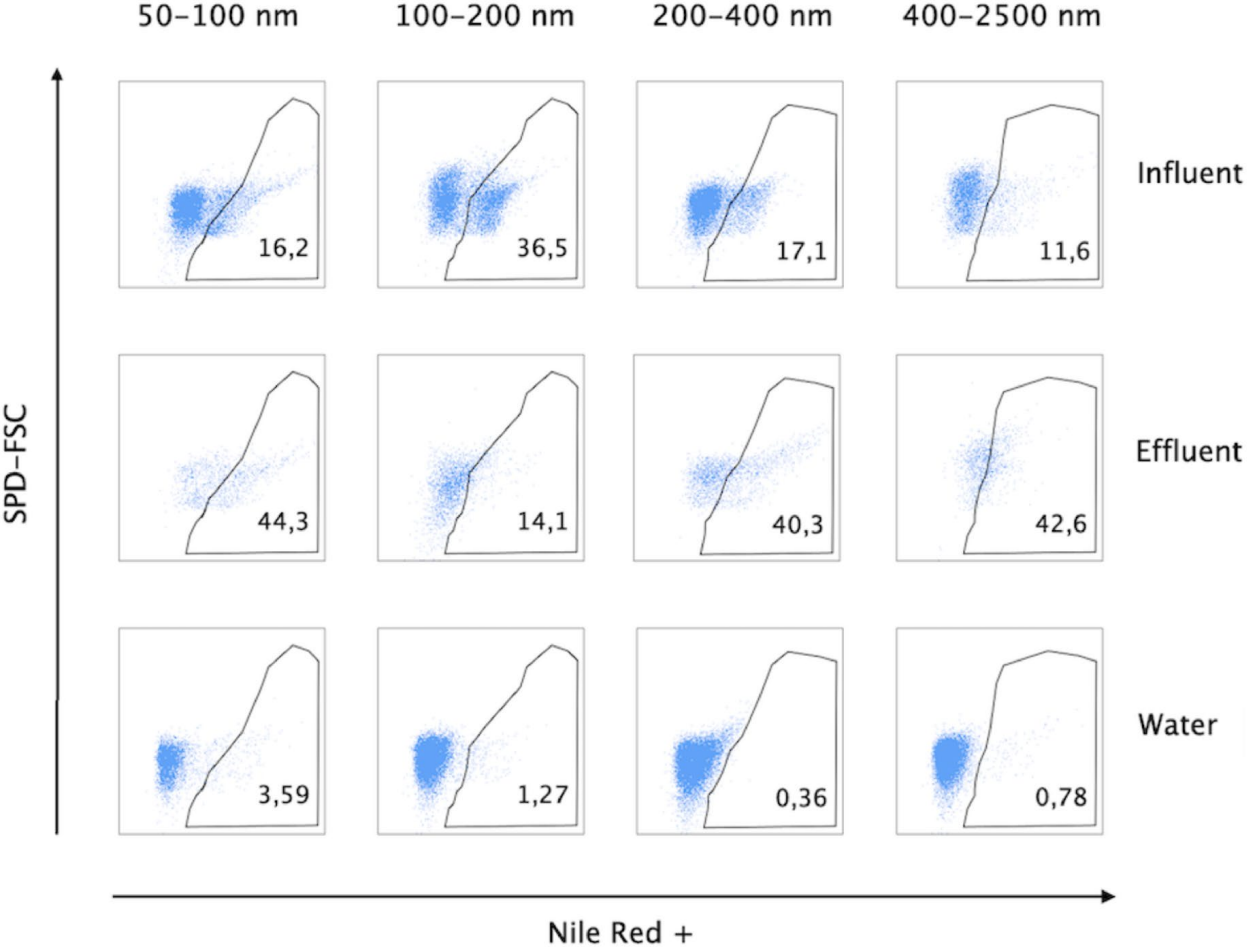
Nanoflow cytometric measurement of the percentage of plastic particles in sewage and treated sewage effluent. Dot plots of forward-scattered light (Y-axes) versus Nile Red fluorescence (X-axes). The nanoparticles were recovered following filtration with a cut-off size indicated at the top of each column. The number inside each plot indicates the percentage of plastic particles out of the total events detected. The region drawn in each plot delimits the area within which an event is considered as plastic particle positive for Nile Red-positive. The control (displayed as Water, bottom panels) corresponds to ultrapure water used as a negative quality control in the experiment. Events shown in blue were recorded above the detection thresholds in the individual experimental samples and negative controls. SPD-FSC: Small particle detector-forward scatter.

The reported abundance (Fig. 2) refers to the percentage of Nile Red–positive events relative to background noise, determined using two detection thresholds. It is expressed as a relative proportion rather than an absolute count. All samples were recorded for the same duration and under identical pressure conditions. The sample flow rate was maintained at 20% of the total system flow, corresponding to 0.2 PSI above the 30 PSI sheath fluid pressure in the flow cytometer. The total event rate, defined as the number of detected particles per second, remained below 2000 events s⁻^1^ prior to threshold application, and below 100 events s⁻^1^ after applying the fluorescence and scatter-based detection thresholds. Because only duplicate composite samples (n = 2) were analysed for each stage, the study was not statistically powered for significance testing; abundance values are therefore given descriptively as mean ± SD.

The effective detection of MPs/NPs in environmental samples using Nile Red staining and fluorescence microscopy was demonstrated, reporting recovery rates as high as 96% for spiked sediment samples, though specific size-resolved recovery values were not detailed^49^. Moreover, another study achieved up to 96% staining efficiency for particles in the 600–15,000 nm range using flow cytometry^52^. Accurate quantification of NPs, particularly those below 1000 nm, remains a significant analytical challenge due to phenomena such as the ‘swarm effect.’ In this study, we observed that particles smaller than 50 nm were prone to being detected as single events, leading to potential underestimation of both particle counts and size distributions. To reduce this error, sequential size fractionation was performed (from 2500 to 1 nm). By analysing each size fraction independently, the impact of the swarm effect was reduced, and the accuracy of particle quantification within each range was improved^53,53^. In addition, the results demonstrate high reproducibility across technical replicates and negative controls, supporting the reliability of the size-fractionated detection method. However, for particles < 100 nm, their quantification remains semi-quantitative. This is due to residual swarm effects and limited binding efficiency of fluorescent dyes at the nanoscale, which can reduce detection sensitivity and introduce additional uncertainty^54^.Scanning Electron Microscopy (SEM) (model Zeiss Merlin, Zeiss, Oberkochen, Germany), was used to analyse the morphology of the recovered plastic particles. Before imaging, the samples were sputter-coated with iridium (3–5 nm) to enhance the conductivity of the sample. The coated samples were then examined under the SEM operating at an accelerating voltage of 2–10 kV. The SEM had a field emission source. Specifically, SEM imaging (Fig. 4) was employed to visualise the morphology and surface features of particles recovered after the extraction process. To confirm the chemical identity of recovered nanoplastics, we conducted confocal Raman spectroscopy on selected particles. This combined approach allowed us to link the visual morphology observed in SEM with molecular-level confirmation from Raman spectra, thereby validating the presence of specific polymer types. Additionally, for the size classification and quantification of NPs, an Invitrogen Bigfoot Spectral Cell Sorter equipped with Sasquatch Software version 1.19.4, (Thermo Fisher Scientific, Waltham, Massachusetts, USA) was used. The instrument was equipped with five lasers (355 nm UV, 405 nm violet, 488 nm blue, 532 nm yellow-green, and 640 nm red), over 40 fluorescence detectors, and five light scatter detectors. A high-sensitivity forward scatter detector (FSC), configured for the 405 nm laser, enabled detection of particles as small as 100 nm. The lasers were calibrated daily during the automated QC process with the Bigfoot™ Calibration Beads (Ref. PL00287) that include a mix of three different calibration beads and three peaks of emission.

### Contamination control

Sample digestion was conducted under fume hoods, with the work surface cleaned using 70% ethanol and covered with aluminium foil to minimise contamination during sampling and analysis. The use of plastic materials was avoided in the experimental work whenever possible, and glass or metal components were used instead. New sealed glass petri dishes (90 mm diameter) were used to avoid cross-contamination. All laboratory equipment and materials, including stainless steel sieves and glassware, were thoroughly cleaned by soaking in concentrated HCl and then rinsed with ultrapure type-1 grade Milli-Q water (MilliporeSigma, Burlington, MA, USA). To prevent cross-contamination, cleaned items were covered with glass lids or aluminium foil before and during all processing steps. The membrane filters used were composed of cellulose acetate and polycarbonate, both commonly employed in MP studies due to their low autofluorescence and minimal leaching risks^55,56^. Blanks were carried out to assess atmospheric deposition in the labs (n = 3) and also throughout the whole analytical process. No Nile Red-positive fragments were found, and only five fibres were detected in the blanks. All liquids were filtered through a 2500 nm cellulose acetate filter (90 mm diameter; Millipore, Burlington, MA, USA) before use, ensuring minimal experimental error due to contamination^57^.

### Analytical quality control

All samples were predigested with 30% H_2_O_2_ (50 °C, 2 h) to eliminate residual neutral lipids. In our matrix-matched set of pre-filtered Milli-Q blanks, this treatment reduced Nile-Red-positive background to ≈ 4% of the signal recorded for influent samples, well within the ≤ 5% blank contribution reported for oxidised wastewater in recent flow-cytometry benchmarks. This confirmed that background artefacts remained within the quality-assured window for optimised- flow cytometry protocols^58,59^.

During nano-flow cytometry the only accepted events were when they satisfied both (i) an SPD-FSC size gate that excluded electronic noise and large aggregates, and (ii) a yellow-orange fluorescence gate centred at 615 ± 7.5 nm. Nile Red bound to hydrophobic plastics emits within this band, whereas the > 650 nm emission produced when Nile Red associates with the polar lipids that survive H_2_O_2_ digestion is automatically rejected^60^.

Confocal Raman random spot-checks of 120 Nile Red-positive cytometry events confirmed over 95% as bona fide polymers, consistent with the accuracy of other high-throughput Nile Red workflows. For instance, one study reported a misclassification rate of only 1% when distinguishing plastics from natural polymers in seafood matrices^61^, while another achieved 95% correct classification (≈ 4% error) in spiked environmental samples^60^. Collectively, these controls constrain any overestimation of particle numbers to ≤ 5%, which is below the sampling variability of the present study.

### Analytical method

Raw sewage and TSE samples were filtered through a 200 nm pore size mixed cellulose acetate membrane filter (47 mm diameter; MilliporeSigma, Burlington, MA, USA). The filtrate was used to determine the chemical oxygen demand (COD), ammonium-nitrogen (NH_4_^+^-N), nitrite-nitrogen (NO_2_–N), nitrate-nitrogen (NO_3_^−^–N), and phosphate as phosphorous (PO_4_^3−^P) using ready-to-use kits (Hach, Düsseldorf, Germany). Biological Oxygen Demand (BOD_5_) was measured following the Standard Methods for the Examination of Water and Wastewater (APHA 2005). These parameters were analysed to evaluate the background organic and nutrient loads in raw and treated wastewater. This contextual information supports the interpretation of particle dynamics and potential interferences during staining and cytometric detection, especially in high-organic matrices.

## Results and discussion

Building on the confirmed specificity of Nile Red fluorescence for plastic particle detection, the approach was applied to assess the presence and size distribution of MPs and NPs in both raw sewage and TSE. As shown in Fig. 2, distinct populations of plastic particles, including small MPs and NPs, were identified in these samples using Nile Red staining in combination with nanoflow cytometry. Nile Red dye specifically stains hydrophobic molecules, such as plastic particles, making it particularly suitable for detecting both MPs and NPs in complex matrices^62^. The nanoflow cytometry analysis showed two distinct populations (Fig. 2): one within the gated region (shown as blue dots within the polygons), corresponding to plastic particles positive for Nile Red, and another outside the gated region (shown as blue dots outside the polygons), consisting of particles negative for Nile Red, such as cell debris or other non-plastic particulates.

In the raw sewage, plastic particles represented in total 16% of the events (particles) recorded in the 50–100 nm size range fraction; 36% in the 100–200 nm fraction; 17% in the 200–400 nm fraction; and 11% in the 400–2500 nm fraction. Overall, in the raw sewage fractions analysed, plastic particles accounted for a median of 16% (± 10%) of the events. The treated effluent contained plastic particles across various size fractions. In the 50–100 nm fraction, plastic particles represented 44% of the total particles, followed by the 100–200 nm fraction, where they accounted for 14% and, in turn, by the 200–400 nm fraction with 40% of plastic particles present. A mix of NPs and small MPs (> 1000 nm) was found in the 400–2500 nm fractions, contributing to 42% of the total plastic particles. Overall, in the treated effluent sewage fractions analysed, plastic particles accounted for a median of 41% (± 13%) of the events. This confirms the presence of NPs and small MPs in TSE and highlights the efficiency of nanoflow cytometry in detecting particles within this size range.

A distinct difference was observed between raw sewage and TSE (Fig. 2). In raw sewage, the majority of Nile Red-positive plastic particles were within the 100–200 nm range, whereas in TSE, most were detected in the < 100 nm range. This shift suggests an increase in red fluorescence intensity in TSE, confirming a higher concentration of NPs in the treated effluent. The proportion of Nile Red-positive particles in the 100–200 nm range increased to 36% in TSE. The highest concentration of Nile Red-positive particles was in the 50–100 nm range, accounting for 44% of total detected events, where an event refers to an individual particle recorded by the instrument. The next most abundant size range was 200–400 nm, comprising 40% of detected particles. These fractions contained significant amounts of plastic, including NPs and MPs. This shift in particle size and abundance between influent and effluent is consistent with dynamic changes occurring during treatment, possibly involving in-plant fragmentation mechanisms, but a causal link cannot be confirmed with the present data.

Nile Red fluorescent probes have been shown to aggregate, potentially leading to false-positive signal detection^38^. Moreover, we needed to exclude the possibility that any residues from the filtration process could be stained by Nile Red, generating false-positive signals. To address this question, we recorded mock samples of ultrapure water for an extended time (5 min) that underwent the same sample preparation process as the wastewater, in order to compare a similar number of events as the most abundant (influent) samples. Background interference, including signals from Nile Red aggregation or dissolved dye, was minimal at ≈ 4% (Fig. 2, control “Water”). Here, “4%” refers to the proportion of background fluorescence signal relative to the total detected fluorescence. This control established a reliable reference for distinguishing plastic-related fluorescence from non-specific signals, confirming the robustness of the detection method.

**Fig. 3:**
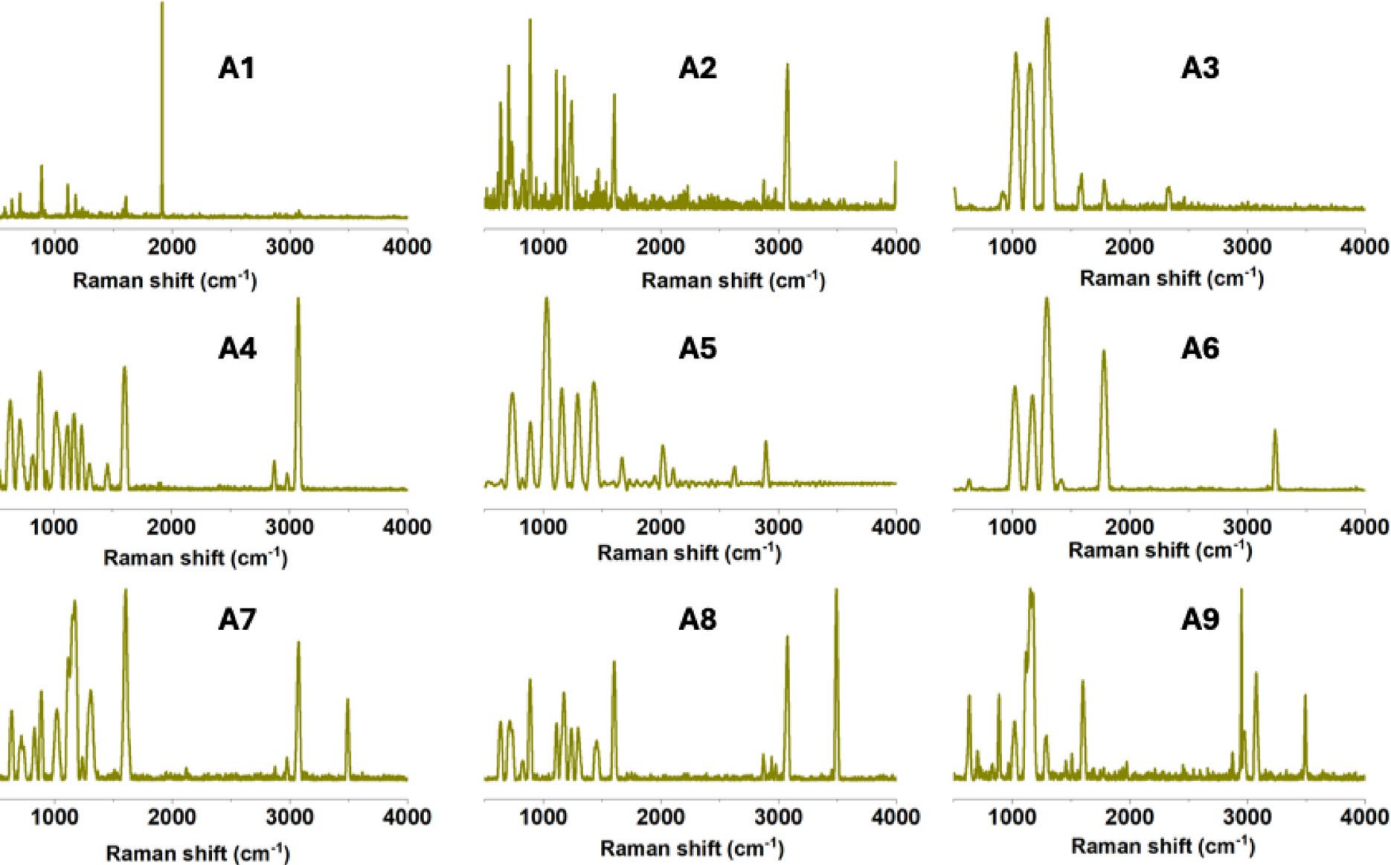
Representative Raman spectra of confirmed plastic particles (500–2000 nm size range) recovered from the study in treated sewage effluent. Spectra shown are baseline-corrected and matched against the ST Japan 6.1 Raman spectral database. Identified polymers and their corresponding Hit Quality Index (HQI) values are A1: polyethylene (HQI: 84.77), A2: polytetrafluoroethylene (HQI: 87.92), A3: polystyrene (HQI: 91.63), A4: polypropylene (HQI: 87.78), A5–A6: nylon (HQI: 96.66), A7-A9: unidentified, showing bands from organic substances.

Differences in recovery rates were observed only between the influent and effluent samples, with the effluent samples exhibiting a lower rate (21.1% on average), as shown in density plots (Fig. 2). The recovery rate of nanoflow cytometry for the different sizes is represented by the percentage numbers shown in the corresponding plots. To further characterise the isolated plastic particles from TSE in the < 2500 nm fraction, confocal Raman spectroscopy was employed. The spectra revealed distinct signals corresponding to common plastic materials (Fig. 3). Multiple plastic types were identified, including PS, PP, PE, polyvinyl chloride (PVC), polytetrafluoroethylene (PTFE), and polyamide (PA or nylon). Despite the enhanced sensitivity of nanoflow cytometry, particularly when selecting the SPD-FSC channel to improve signal resolution, detecting NPs smaller than 100 nm remains a challenge. Particles smaller than 50 nm scatter very little light with low intensity and have too little surface area to load a sufficient amount of dye for reliable detection. For these reasons, they are easily confused with the electronic noise of the instrument and background particles, even from filtered buffers and clean samples. Moreover, multiple particles smaller than 50 nm may be detected as one, altering counts and sizing, due to the “swarm effect”^54^. As a result, 50–100 nm bins are treated as semi-quantitative and therefore discussed only in relative terms instead of absolute numbers.

Particles ≥ 50 nm could be detected. However, confocal Raman spectroscopy could not chemically identify all NPs, particularly in the < 300 nm size range, due to its diffraction-limited resolution. In addition, while Nile Red staining enabled efficient detection through nanoflow cytometry, it may introduce false positives in complex wastewater matrices due to non-specific interactions with residual biogenic material. To reduce this uncertainty, future work should explore alternative dyes such as BODIPY and Coumarin-6, which offer greater selectivity and signal stability. Moreover, cross-validation using orthogonal techniques will be essential to confirm NP identity. Although established methods like pyrolysis-GC/MS can provide bulk polymer identification, they lack spatial resolution and particle-level context.

Previous studies have reported the presence of plastics such as PTFE, PE, PP, PA, high- or low-density PE, and polyacrylamide (PAM) in wastewater, including raw sewage and TSE, often linked to indoor sources like textiles and commercial products^63–65^. In this study, similar NPs, including PTFE, PS, and PE, were consistently detected in TSE. Initially, it was thought that possible contamination from lab materials, such as glassware, filters, air, or water used during the analysis, may have occurred. However, the blanks, which consisted of ultrapure water that underwent the same preparation steps, including nanofiltration and staining, implemented to eliminate these sources of contamination, did not contain such particles.

**Fig. 4:**
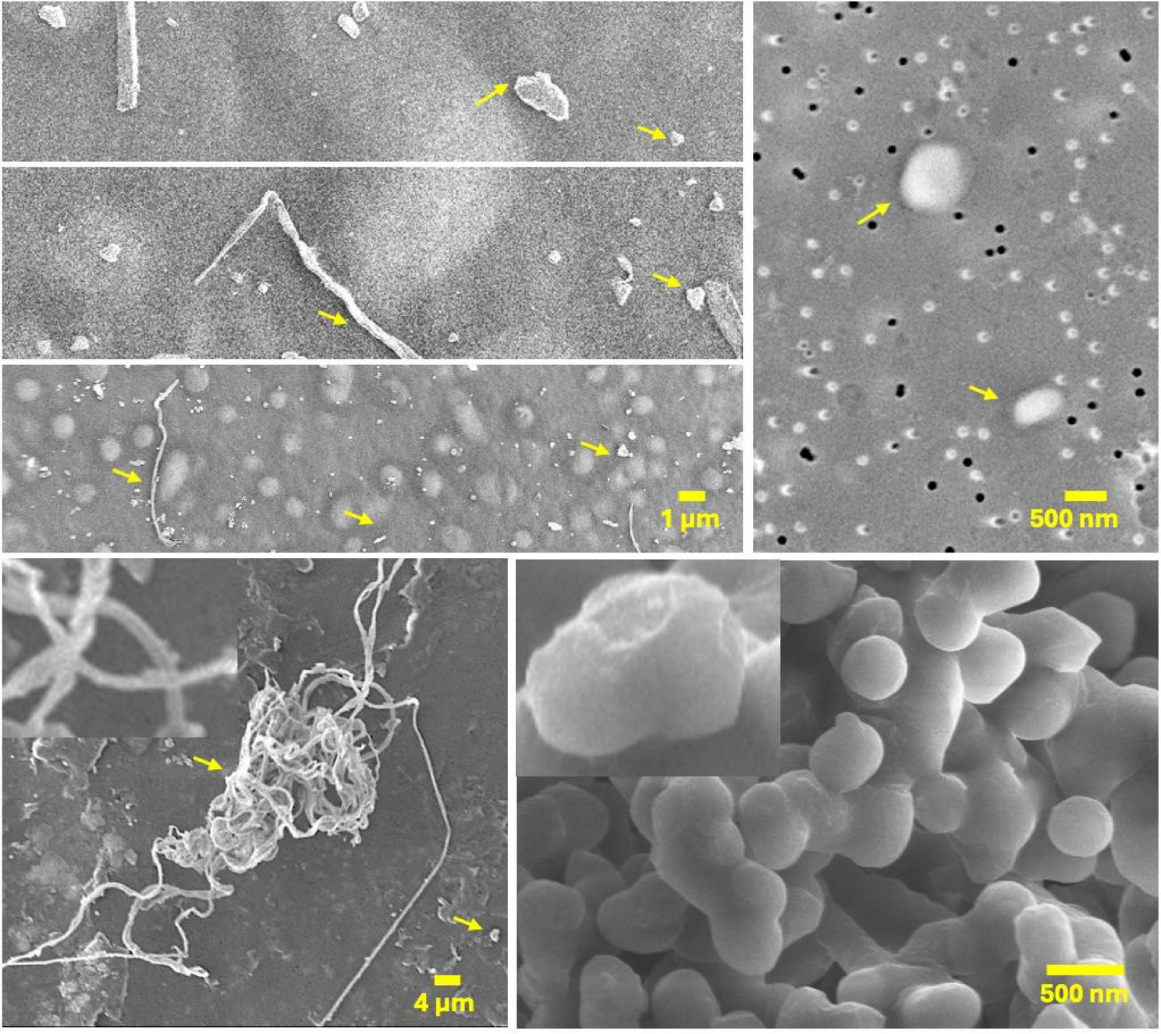
Scanning electron micrographs of plastic particles recovered from treated sewage effluent.

The morphology of recovered NPs from TSE (Fig. 4) included irregularly shaped and spherical particles, consistent with previous reports on wastewater MPs^32^. The persistence of these particles in TSE from a full-scale activated sludge process raises significant environmental concerns, as they can evade filtration and enter natural water bodies, contributing to ecological damage. Literature highlights the risk of MPs accumulating in aquatic ecosystems, impacting marine life and the food web^66^. The fractionation and persistence of polymers like PS, PE, PP, and PTFE in the treatment system are strongly influenced by their intrinsic properties. A recent study demonstrated that factors such as polymer density, particle morphology, surface hydrophobicity, and chemical stability determine whether particles are retained in sludge or escape with the effluent^67^. Low-density polymers like PE and PP often evade settling but may eventually be removed via aggregation and biofouling, while denser polymers such as PS and PTFE are more likely to settle early and resist degradation. These mechanisms help explain the variable retention observed in different treatment setups and underscore the need to design processes that target the specific behaviours of diverse MPs and NPs. The findings highlight the urgent need to integrate NP-specific treatment methods into wastewater management systems. Future studies should focus on expanding the analysis to include full-scale wastewater treatment plants employing different technologies, and not just the activated sludge process, such as the aerobic granular sludge process, membrane bioreactors, and moving bed biofilm reactors, with the aim of understanding the magnitude of NP prevalence in the effluent of treatment plants and the factors that could help in their retention or removal within the plant.

## Conclusion

This study confirmed the presence of NPs and small MPs in both raw sewage and TSE from an activated sludge-based sewage treatment system in Saudi Arabia. Nanoflow cytometry, combined with Nile Red staining, was effective in detecting and quantifying plastic particles, particularly those ≤ 1000 nm. Results demonstrated that despite biological treatment, NPs persist in TSE, representing 40% (± 13.4%) of all particles detected in the 50–400 nm size range. This suggests that the activated sludge process is ineffective at removing plastics and may even increase their concentration. Furthermore, plastic particles accounted for 16% (± 10%) of total events in raw sewage, increasing to 41% (± 13%) in treated effluent, highlighting the limited efficiency of conventional wastewater treatment in eliminating plastic contaminants. Various plastic types, including PS, PE, and PP, were identified in both raw sewage and TSE. Confocal Raman spectroscopy successfully identified plastics in the 300–2500 nm size range but failed to detect NPs smaller than 300 nm due to limitations in resolution. This study shows that effluents from wastewater treatment plants that carry out secondary treatment have a high percentage of particles in the NPs range and lower MP size range, where the abundance of particles identified as plastic increases with decreasing particle size. These findings highlight the need for incorporating NP-targeted removal technologies in wastewater treatment and emphasise the importance of regulatory monitoring of NPs in effluents, especially in water-scarce regions relying on effluent reuse. To support such monitoring, future globally standardised protocols should include a defined particle size range (e.g., 50– < 1000 nm) for NPs, validated detection limits, reproducible analytical workflows, and harmonized reporting formats, criteria increasingly recognized as essential for reliable NP assessment in complex matrices.

## Supplementary Information

Below is the link to the electronic supplementary material.

Supplementary Material 1

## Data Availability

Data will be available upon request. Please contact Mrs Shaima Iskandarani (shaima.iskandarani.22@ucl.ac.uk) to request the data.
