# Supplementary material for: Characterization of nanoplastics and small-sized microplastics in sewage treatment
Source: Sci Rep. 2025 Aug 17;15:30089. doi: 10.1038/s41598-025-15504-9 (PMC12358546; doi:10.1038/s41598-025-15504-9)
Supplement: Supplementary file 1 — Supplementary Material 1 [file 41598_2025_15504_MOESM1_ESM.docx]

**Supplementary Materials**

a

b


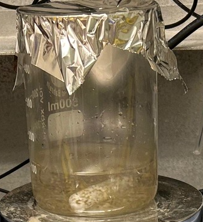

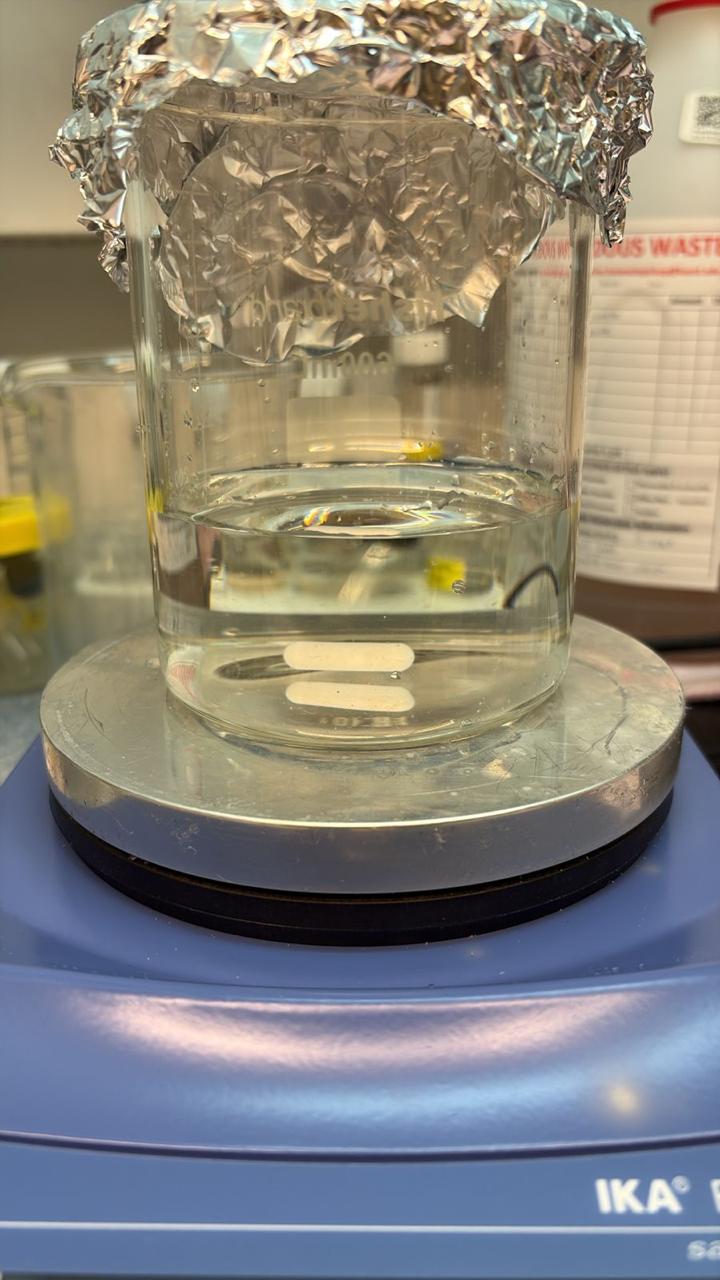


a

b

**Figure S1. Representative images of different samples taken at various stages of the digestion process, captured for laboratory work documentation purposes.**

(a) A sample during hydrogen peroxide digestion, showing visible suspended solids and organic matter.

(b) A digested effluent sample after treatment, showing significantly reduced suspended solids prior to Nile Red staining.

**Note S1. Estimation of Plastic Particle Abundance (particles/L)**

Plastic particle abundance in raw sewage and treated sewage effluent was estimated based on gated cytometry event counts and sample volumes processed.

Cytometry Parameters (from Methods):

Sample volume analyzed per run: 2 mL

Event rate with plastic-specific gating: ~75 events/sec

Acquisition time: 60 seconds

Total events analyzed per sample: ~4,500

Plastic-positive event percentages:

Raw sewage (influent): 16.65%

Treated sewage effluent (TSE): 41.45%

Abundance Calculations:

Raw sewage:

Plastic events = 0.1665 × 4,500 = 750 particles in 2 mL

→ 375 particles/mL = 3.75 × 10⁵ particles/L

Treated effluent:

Plastic events = 0.4145 × 4,500 = 1,865 particles in 2 mL

→ 932.5 particles/mL = 9.33 × 10⁵ particles/L
